# An association study of *Taq1A ANKK1* and *C957T* and − *141C DRD2* polymorphisms in adults with internet gaming disorder: a pilot study

**DOI:** 10.1186/s12991-017-0168-9

**Published:** 2017-12-08

**Authors:** Soo-Hyun Paik, Mi Ran Choi, Su Min Kwak, Sol Hee Bang, Ji-Won Chun, Jin-Young Kim, Jihye Choi, Hyun Cho, Jo-Eun Jeong, Dai-Jin Kim

**Affiliations:** Department of Psychiatry, Seoul St. Mary’s Hospital, The Catholic University of Korea College of Medicine, Seoul, 222, Banpo-daero, Seocho-gu, Seoul, 06591 South Korea

**Keywords:** Internet gaming disorder, Dopamine D2 receptor, ANKK1, TaqMan assay, Personality and temperament

## Abstract

**Background:**

Though Internet gaming disorder (IGD) is considered to share similar genetic vulnerability with substance addictions, little has been explored about the role of the genetic variants on IGD. This pilot study was designed to investigate the association of the *Taq1A* polymorphism of the ankyrin repeat and kinase domain containing 1 (*ANKK1*) gene and *C957T* and − *141C* of the dopamine D2 receptor (*DRD2*) with IGD and their role on the personality and temperament traits in IGD among adult population.

**Methods:**

Sixty-three subjects with IGD and 87 control subjects who regularly played Internet games were recruited. Self-administered questionnaires on self-control, dysfunctional impulsivity, and temperament and character domains were done. The *Taq1A ANKK1* and the *C957T* and − *141C ins/del* from the *DRD2* genes were genotyped using the specific TaqMan PCR assay.

**Results:**

The distributions of allele and genotype frequencies were not significantly different between the IGD and control groups in both genders. In male, excessive gaming and use of gaming to escape from a negative feeling were associated with the *del−* genotype of the − *141C*. Among IGD, the *del*+ genotype was associated with higher novelty seeking. Logistic regression showed no predictive value of these polymorphisms for IGD when using age and gender as covariates.

**Conclusions:**

Though no direct association of the *Taq1A ANKK1* and *C957T DRD2* variants with IGD were observed, the − *141C* polymorphism may play a role in IGD via mediating symptoms or temperament traits.

**Electronic supplementary material:**

The online version of this article (10.1186/s12991-017-0168-9) contains supplementary material, which is available to authorized users.

## Introduction

Internet gaming disorder (IGD) is conceptualized as a behavioral addiction that excessive preoccupation and loss of control over Internet gaming eventually leads to the functional impairment [1]. Growing evidence suggested that IGD resembles substance additions in phenomenology, genetic and environmental risk factors, and neurobiological mechanisms [2]. Individuals with IGD showed impulsivity and response disinhibition in Go/No-Go tasks [3], tendency to make risky decision in Cups tasks [4], and increased craving when gaming-related cues were presented [5]. Furthermore, dysregulation of the dopamine D2 receptors in the striatum was observed in Internet addiction [6]. On account of this, a previous study revealed the association of the *Taq1A* polymorphism of the ankyrin repeat and kinase domain containing 1 (*ANKK1*) gene, which was associated with altered availability of dopamine D2 receptor, with IGD in male adolescents [7]. Family and twin studies have shown that the genetic factors accounted for 41–66% of the variance of the Internet addiction [8–10], which was comparable to that of substance addictions [11]. Accordingly, the identification of genetic vulnerabilities is considered as providing better understanding of the neurobiology of IGD and improving intervention outcomes. However, little has been explored on the role of the genetic variants on IGD.

The genetic polymorphisms that potentially alter the availability and expression of the dopamine D2 receptor have been known to be associated with both substance and behavioral addictions [12, 13]. The *Taq1A* polymorphism (SNP ID: rs1800497), which is located in exon 8 of *ANKK1*, adjacent to the terminal codon of the *DRD2* (OMIM*126450; gene map locus 11q23.2), is known to alter binding specificity and reduce DRD2 expression in striatum and associated structures. A significant number of genetic studies have found the association of the *Taq1A* polymorphism with substance addiction and impulsivity and psychopathic traits in alcohol dependence [14–16]. The *C957T* polymorphism (SNP ID: rs1799732), which is located on exon 7 of the *DRD2* (gene map locus 11q23), is known to alter DRD2 availability and be associated with alcoholism with higher prevalence of the CC genotype [17, 18]. The − *141C Ins/Del* polymorphism (SNP ID: rs6277), which is located on promoter of the *DRD2* (gene map locus 11q23), alters striatal D2 receptor binding potentials, and is associated with alcoholism [16, 19, 20]. Given the gene performance and the association with substance addiction, the three polymorphisms may play a crucial role in IGD. To date, however, no study has investigated the association of these polymorphisms with IGD.

Personality and temperament traits such as high neuroticism, psychoticism, sensational seeking, and reward dependence and low self-directedness and cooperativeness were associated with IGD [21–24]. Personality traits are strongly influenced by genetics. Twin studies which reported individual differences in personality showed about 50% of heritability, which means that about half of the variation in personality traits is affected by genetics [25, 26]. Though this assumption is oversimplified and several studies performed in healthy volunteers have reported no association between the variants of *DRD2* gene and personality and temperament traits [27, 28], relations were found in substance and behavioral addiction. For example, the *Taq1A* A1 carriers of pathological gambling had higher harm avoidance and lower self-directedness than non-A1 carriers, and the *C957T T* carriers of alcohol dependence had higher psychopathic tendency [18, 29]. Given that genetics could influence addictive disorders either directly or mediated via personality traits [27], genetics may better account for the vulnerability for IGD when personality and character traits are taken into account. At present, two studies have investigated the association of genetic variants with temperament traits in excessive Internet users: the *Taq1A* polymorphism with higher reward dependence and the short allelic variant of the serotonin transporter gene (SS-5HTTLPR) with higher harm avoidance [7, 30]. Despite, these studies only included male adolescents who normally manifested higher sensation seeking and risk taking behavior, used rather subjective definition of excessive Internet users, and represented only personality dimensions of interest. Accordingly, a need to investigate the genetics of IGD with consideration of the temperament and personality traits in adults, who have more stable personality structure than adolescents, is emerging.

The present pilot study was designed to investigate the association of the genetic polymorphisms of *DRD2* (− *141C* and *C957T*) and *ANKK1* (*Taq1A*) with IGD and its symptoms, and the role of these polymorphisms on IGD and various personality and temperament traits of IGD.

## Materials and methods

### Participants

We recruited volunteers who agreed with and were able to follow the study design from three online surveys conducted in 2015 and 2016 regarding the Internet gaming behaviors. All participants were assessed using the diagnostic criteria for IGD of the Diagnostic and Statistical Manual of Mental Disorder, 5th edition [31]. The IGD group was defined by endorsement of at least five or more of the nine criteria over a 12-month period. The control subjects were determined as individuals who played the Internet games regularly but were not considered as having IGD. All participants aged 19 or over and had at least 12 years of education.

We interviewed all subjects for the history of neurological or severe medical illness and assessed intellectual function using the Korean version of the Wechsler Adult Intelligence Scale version IV (K-WAIS-IV) to exclude intellectual disability. After excluding four subjects with intellectual disability and one with a history of neurological illness (brain tumor, postoperative status), 63 IGD and 87 control subjects were included in this study. The general characteristics were as follows: age range of 19–47 years old; mean age 30.09 years [standard deviation (SD): 6.343]; number of male participants 115 (76.7%); time spent gaming on weekdays 2.03 h (SD: 1.35); and time spent gaming on weekends 3.28 h (SD: 1.94).

The study protocol was approved by Institutional Review Boards of Seoul St. Mary’s Hospital (IRB number: KC15EISI0103). This study met the ethical standards of the Declaration of Helsinki, including obtaining informed consent from all participants and adhering to the privacy rights of participants.

### Measures

Participants were asked to fill out questionnaires regarding personality and temperament traits as follows: the brief self-control scale (BSCS), the Dickman dysfunctional impulsivity inventory (DII), and the temperament and character inventory-revised short (TCI-RS). Questions about time spent gaming on weekdays and weekends were asked separately.

The BSCS measures the ability to override or change one’s inner response as well as to interrupt undesired behavioral tendencies and refrain from acting on them [32]. The BSCS is a 13-item questionnaire with a 5-point Likert scale, and higher scores indicate lower self-control.

The DII is composed of 12 out of 23 items of the Dickman functional and dysfunctional inventory [33]. In DII, dysfunctional impulsivity refers to the tendency to act with less forethought than most people of equal ability when this tendency is a source of difficulty. Items are rated true (1) or false (0) and higher scores indicate higher dysfunctional impulsivity.

The temperament and character inventory (TCI) has been widely used in the investigations of human psychobiological behaviors [34]. TCI measures four temperament dimensions: novelty seeking (NS), harm avoidance (HA), reward dependency (RD), and persistence (*P*), and three character dimensions: self-directedness (SD), cooperativeness (*C*), and self-transcendence (ST). We used a shortened TCI-R inventory (TCI-RS), which was consisted of 140 questions [35]. Items on the TCI-RS are rated on a 5-point Likert scale, and higher scores indicate higher prominence of the dimensions.

### Genotyping: a specific TaqMan PCR assay

DNA was isolated from the whole blood using a Wizard Genomic DNA Purification Kit (Promega, Madison, WI, USA) according to the manufacturer’s instructions. Genotypes for *Taq1A* (SNP ID: rs1800497), − *141C Ins/Del* (SNP ID: rs1799732), and *C957T* (SNP ID: rs6277) polymorphic loci were assessed using the TaqMan SNP genotyping assays (Thermo Fisher Scientific, Carlsbad, CA, USA) on a ViiA 7 Real-Time PCR System (Thermo Fisher Scientific, Foster City, CA, USA). All genotypes were reported using the allelic discrimination program in QuantStudioTM Real-Time PCR Software v1.1 (Thermo Fisher Scientific, Foster City, CA, USA). Only one − *141C* genotype of a male control subject was missing. After genotyping, the *Taq1A* was grouped as the A1+ (homozygous and heterozygous for the A1 allele) and the A1− genotypes (homozygous for the A2 allele), the *C957T* as the T+ (homozygous and heterozygous for the T allele) and the T− genotypes (homozygous for the C allele), and the − *141C Ins/Del* as the *del*+ (homozygous and heterozygous for the deletion allele) and *del* *−* genotypes (homozygous for the insertion allele).

### Statistical analysis

*χ* ^2^ tests were done to investigate the association of polymorphisms with IGD and its symptoms. Independent *t* tests were carried out to compare the clinical and personality variables between the IGD and control groups except the TCI-RS. Analyses of covariance (ANCOVA) were performed using age as a covariate to explore the TCI-RS profiles between groups since TCI may vary with age [36]. When age had significant interactions with the TCI variables, the results of the independent *t* tests were present. Subgroup analyses in the IGD group were performed to investigate the associations of the polymorphisms with temperament and personality predispositions in IGD. Binary logistic regression analysis was conducted to calculate the predictive value of each genotype using age and gender as covariates and odds ratio (OR) and 95% confidential interval (CI) were present. *χ* ^2^ goodness-of-fit tests were performed in order to calculate the correspondence between the observed number of homozygous and heterozygous individuals and the numbers expected based on Hardy–Weinberg equilibrium. All statistical analyses were performed using SPSS 24.0 (SPSS Inc., Chicago, IL, USA).

## Results

### Clinical characteristics and personality traits

As shown in Table 1, participants with IGD were older (IGD: 32.03 ± 6.528, control: 28.68 ± 5.812, *t* = − 3.398, *p* = .001) and had higher prevalence of male (IGD: 87.4%, control: 61.9%, *χ* ^2^ = 13.232, *p* = .000). IGD spent more time on gaming both weekdays and weekends and had higher scores on the BSCS and DII, indicating lower self-control ability and higher dysfunctional impulsivity, than the control subjects. As for the TCI-RS, the scores of NS, *P,* and ST were higher in IGD in independent *t* tests. However, since these three variables had significant interaction effect between group and age, ANCOVA was not performed for them (see Additional file 1 for the scatter plot of ANCOVA and regression slope of NS, *P*, and ST). Other TCI domains showed no significant differences between groups in ANCOVA. Additional subgroup analyses which divided the sample into two groups (age < 30 vs age ≥ 30) showed no significant differences in TCI-RS domains between groups (results not shown).

**Table 1:** Comparisons of clinical and personality variables

	Control (*n* = 87)	IGD (*n* = 63)	*χ* ^2^/*t*/*F*	*P*
Age (years old)	*28.58* *±* *5.812*	*32.03* *±* *6.528*	*−* *3.398*	*.001*
Male proportion (%)	*76 (87.4%)*	*39 (61.9%)*	*13.232*	*.000*
Weekday gaming hours	*1.66* *±* *1.19*	*2.57* *±* *1.40*	*−* *2.805*	*.007*
Weekend gaming hours	*2.88* *±* *1.88*	*3.86* *±* *1.90*	*−* *2.055*	*.044*
BSCS	*36.17* *±* *6.547*	*41.44* *±* *5.482*	*−* *5.196*	*.000*
DII	*4.16* *±* *3.429*	*6.57* *±* *2.988*	*−* *4.498*	*.000*
Novelty seeking	*31.59* *±* *8.997*	*37.54* *±* *8.561*	*−* *2.689* ^a^	*.009*
Harm avoidance	33.104 ± 2.071	33.666 ± 1.642	.044	.834
Reward dependence	42.046 ± 1.355	44.967 ± 1.709	1.760	.189
Persistence	*43.05* *±* *10.538*	*49.46* *±* *8.659*	*−* *2.595* ^a^	*.012*
Self-directedness	49.368 ± 1.371	50.727 ± 1.730	.372	.544
Cooperativeness	52.759 ± 1.448	55.534 ± 1.827	1.391	.243
Self-transcendence	*18.51* *±* *8.775*	*24.92* *±* *10.311*	*−* *2.725* ^a^	*.008*

*IGD* Internet gaming disorder, *BSCS* brief self-control scale, *DII* Dickman dysfunctional impulsivity scale

* *p* < .05, ** *p* < .005

^a^Results of *t* tests

### Association of polymorphisms with IGD and its symptoms

Genotype and allele distributions of three polymorphisms were not significantly different between the IGD and control groups (Table 2). The results were similar when analyzed male and female separately. The observed frequencies of homozygous, heterozygous, and non-carriers of the minor alleles were in Hardy–Weinberg equilibrium (Taq1A ANKK1 SNP: *χ* ^2^ = 0.44, *p* = .51; C957T DRD2: *χ* ^2^ = 0.84, *p* = .36, − *141C* DRD2: *χ* ^2^ = 0.74, *p* = .39).

**Table 2:** Allele and genotype frequencies of ANKK1 and DRD2 polymorphisms

Genotype	ANKK1 Taq 1A			DRD2 C952T			DRD2 − 141C Ins/Del		
	A1A1	A1A2	A2A2	TT	TC	CC	Del/Del	Del/Ins	Ins/Ins
Whole									
Control (*n* = 87)	15 (17.2)	43 (48.3)	30 (34.5)	0 (0.0)	15 (17.2)	72 (82.8)	1 (1.2)	29 (33.7)	56 (65.1)
IGD (*n* = 63)	12 (19.0)	26 (41.3)	25 (39.7)	0 (0.0)	6 (9.5)	57 (90.5)	2 (3.2)	17 (27.0)	44 (69.8)
	*χ* ^2^ = .731, *p* = .694			*χ* ^2^ = 1.808, *p* = .179			*χ* ^2^ = 1.386, *p* = .500		
Male									
Control (*n* = 76)	14 (18.4)	35 (46.1)	27 (35.5)	0 (0.0)	12 (15.8)	64 (84.2)	1 (1.3)	26 (34.7)	48 (64.0)
IGD (*n* = 39)	7 (17.9)	17 (43.6)	15 (38.5)	0 (0.0)	4 (10.3)	35 (89.7)	0 (0.0)	8 (20.5)	31 (79.52)
	*χ* ^2^ = .099, *p* = .952			*χ* ^2^ = .659, *p* = .417			*χ* ^2^ = 3.132, *p* = .209		
Female									
Control (*n* = 11)	1 (9.1)	7 (63.6)	3 (27.3)	0 (0.0)	3 (27.3)	8 (72.7)	0 (0.0)	3 (27.3)	8 (72.7)
IGD (*n* = 24)	5 (20.8)	9 (37.5)	10 (41.7)	0 (0.0)	2 (8.3)	22 (91.7)	2 (8.3)	9 (37.5)	13 (54.2)
	*χ* ^2^ = 2.155, *p* = .341			*χ* ^2^ = 2.210, *p* = .137			*χ* ^2^ = 1.580, *p* = .454		

	ANKK1 Taq 1A		DRD2 C952T		DRD2 − 141C Ins/Del	
Allele	A1+	A1−	T+	T−	Del+	Del−
Whole						
Control (*n* = 87)	57 (65.5)	30 (34.1)	15 (17.2)	72 (82.8)	30 (34.5)	57 (65.5)
IGD (*n* = 63)	38 (60.3)	25 (39.7)	6 (9.5)	57 (90.5)	19 (30.2)	44 (69.8)
	*χ* ^2^ = .425, *p* = 514		*χ* ^2^ = 1.808, *p* = .179		*χ* ^2^ = .368, *p* = .544	
Male						
Control (*n* = 76)	49 (64.5)	27 (35.5)	10 (16.9)	49 (83.1)	27 (36.0)	48 (64.0)
IGD (*n* = 39)	24 (61.5)	15 (38.5)	2 (5.6)	34 (94.4)	8 (20.5)	31 (79.5)
	*χ* ^2^ = .096, *p* = .757		*χ* ^2^ = .659, *p* = .417		*χ* ^2^ = .2.893, *p* = .089	
Female						
Control (*n* = 11)	15 (51.7)	14 (48.3)	3 (27.3)	8 (72.7)	3 (27.3)	8 (72.7)
IGD (*n* = 24)	15 (55.6)	12 (44.4)	2 (8.3)	22 (91.7)	11 (45.8)	13 (54.2)
	*χ* ^2^ = .669, *p* = .413		*χ*2 = 2.210, *p* = .137		*χ* ^2^ = 1.083, *p* = .298	

*IGD* Internet gaming disorder

The association of each genotype with symptoms of IGD was not evident in whole sample (Additional file 2). Subgroup analyses performed in male showed that male with the *del*− genotype showed higher prevalence of the presence of G6 (Continued excessive use of Internet games despite knowledge of psychosocial problems) and G8 (Use of Internet games to escape or relieve a negative mood) than male with the *del*+ (Table 3). No association of the genotypes with IGD symptoms was found in female.

**Table 3:** Association of genotypes with symptoms of IGD in male

	A1+ (*n* = 73)	A1− (*n* = 42)	T+ (n = 16)	T− (*n* = 99)	Del+ (n = 35)	Del− (*n* = 79)
G1	20 (27.4%)	12 (28.6%)	4 (25.0%)	28 (28.3%)	8 (22.9%)	24 (30.4%)
	*χ* ^2^ = .018, *p* = .892		*χ* ^2^ = .074, *p* = .786		*χ* ^2^ = .680, *p* = .410	
G2	16 (21.9%)	12 (28.6%)	3 (18.8%)	25 (25.3%)	6 (21.4%)	22 (27.8%)
	*χ* ^2^ = .641, *p* = .423		*χ* ^2^ = .316, *p* = .574		*χ* ^2^ = 1.500, *p* = .221	
G3	22 (30.1%)	14 (33.3%)	3 (18.8%)	33 (33.3%)	8 (22.9%)	28 (35.4%)
	*χ* ^2^ = .127, *p* = .722		*χ* ^2^ = 1.362, *p* = .243		*χ* ^2^ = 1.778, *p* = .182	
G4	27 (37.0%)	18 (42.9%)	7 (43.8%)	38 (37.4%)	11 (31.4%)	34 (43.0%)
	*χ* ^2^ = .386, *p* = .535		*χ* ^2^ = .167, *p* = .683		*χ* ^2^ = 1.368, *p* = .242	
G5	24 (32.9%)	19 (45.2%)	7 (43.8%)	36 (36.4%)	10 (28.6%)	33 (41.8%)
	*χ* ^2^ = 1.740, *p* = .187		*χ* ^2^ = .321, *p* = .571		*χ* ^2^ = 1.779, *p* = .180	
G6	21 (28.8%)	13 (31.0%)	5 (31.3%)	29 (29.3%)	*6 (17.1%)*	*28 (35.4%)*
	*χ* ^2^ = .061, *p* = .805		*χ* ^2^ = .025, *p* = .874		*χ* ^*2*^ *=* *3.881, p* *=* .049*	
G7	27 (37.0%)	18 (42.9%)	5 (31.3%)	40 (40.4%)	*11 (31.4%)*	*34 (43.0%)*
	*χ* ^2^ = .386, *p* = .535		*χ* ^2^ = .485, *p* = .486		*χ* ^*2*^ *=* *.368, p* *=* *.242*	
G8	20 (27.4%)	15 (35.7%)	5 (31.3%)	30 (30.3%)	*6 (17.1%)*	*29 (36.7%)*
	*χ* ^2^ = .871, *p* = .351		*χ* ^2^ = .006, *p* = .939		*χ* ^*2*^ *=* *4.364, p* *=* .037*	
G9	14 (19.2%)	10 (23.8%)	2 (12.5%)	22 (22.2%)	4 (11.4%)	20 (25.3%)
	*χ* ^2^ = .346, *p* = .556		*χ* ^2^ = .788, *p* = .375		*χ* ^2^ = 2.815, *p* = .093	

G1–G9 represented DSM-5 IGD criteria (Additional file 2)

* *p* < .05

### Role of ANKK1/DRD2 polymorphisms on IGD and personality and temperament predispositions

In the IGD group, the *t* tests showed the association of the *del*+ of the − 141C with higher NS (*del*+: 44.71 ± 9.050, *Del*−: 34.86 ± 6.863, *t* = − 2.973, *p* = .007) (Additional file 3). However, ANCOVA was inapplicable for the investigation of the association of the *del* genotype and NS due to the significant interaction effect (Additional file 1). In subgroup analyses (age < 30 vs. age ≥ 30), no significant association between genotypes and TCI in the IGD group was found. The *Taq1A* and *C957T* polymorphisms were not significantly associated with personality and temperament predispositions in IGD.

Table 4 showed results of the binary logistic regression to predict the risk for IGD using age, gender, and each genotype as covariates. Overall classification accuracy was 72.3% and Nagelkerke R square was 23.0%. Being male (OR = 4.205, 95% CI 1.776–9.956, *p* = .001) and older age (OR = 1.098, 95% CI 1.034–1.166, *p* = .002) were considered as risk factors for IGD. However, any genotype had no predictive value for being IGD.

**Table 4:** Predictive values for Internet gaming disorder

Variables	*B*	S.E.	OR	95% CI	*p*
Male gender	*1.436*	*.440*	*4.205*	*1.776–9.956*	.001**
Age	*.094*	*.031*	*1.098*	*1.034–1.166*	.002**
A1+ of Taq1A ANKK1	− .534	.399	.586	.268–1.281	.180
T+ of C957T DRD2	− 1.161	.607	.559	.255–1.225	.146
Del+ of − 141C DRD2	− 2.797	.950	.313	.095–1.028	.056

*CI* confidential interval

** *p* < .005, * *p* < .05

## Discussion

The purpose of this preliminary study was to explore the role of the *Taq1A ANKK1 and* − *141C Ins/Del* and *C957T DRD2* polymorphisms on IGD. Our results suggested that though these polymorphisms had no association with occurrence and the personality and temperament predispositions in adults with IGD, some symptoms of IGD were associated with the − *141C Ins/Del* polymorphism in male population.

When compared between the IGD and control groups, IGD subjects had unique clinical and TCI characteristics. First, IGD subjects had higher mean age. The prevalence of IGD across different age groups in adult population has not been investigated before. Given that characteristics of gaming behavior were different across age groups in adults [37], adults with IGD may have unique psychological antecedents that are different from those of adolescents IGD. Second, IGD subjects showed high persistence and self-transcendence as well as low self-control and high impulsivity and novelty seeking as reported before [7, 38–40]. Given that high persistence was associated with perfectionism and obsessive–compulsive disorder [36, 41], this may support the compulsive nature of IGD [42]. High self-transcendence may pose the possibility of psychopathology in IGD [43, 44]. On account of the distribution of allele distribution, our sample was nearly absent in the T allele of *C957T* and the del of − *141C DRD2*. According to the refSNP database (https://www.ncbi.nlm.nih.gov/projects/SNP/snp_ref.cgi?rs=1799732 and https://www.ncbi.nlm.nih.gov/projects/SNP/snp_ref.cgi?rs=6277), the prevalence of the T and del allele was quite low in East Asia, ranging from 0.0487 to 0.0625 and 0.136, which were comparable to that of our sample, 0.0747 and 0.209, respectively.

We found that none of the polymorphisms were associated to the occurrence of IGD, which was inconsistent with a previous finding in male adolescents with IGD [7]. Given that adolescents are more eager to take risks and seek for novelties and thus have been considered to be more susceptible to IGD [45, 46], adolescents with risky genetic factors may be more vulnerable to become IGD while adults may be more affected by the complex interaction of genetic and environmental factors. Meanwhile, males with the *del*− genotype more frequently used Internet games excessively despite knowledge of psychosocial problems and to escape or relieve a negative mood. The *del*− genotype was associated with alcoholism, especially in male population, and alcoholic patients with the *del*− continued to drink despite they had protective ALDH2*2 and ADH1B*2 alleles [20, 47]. Meanwhile, the role of the − *141C* polymorphism in vivo is controversial; one study showed healthy volunteers with the *del*+ had higher striatal D2 receptor binding potential while the other showed no significant differences between the *del*+ and *del*− [19, 48]. It can be inferred that the − *141C DRD2* polymorphism may influence specific behaviors observed in addictive disorders, such as excessive use and compensation of negative mood. Interestingly, this finding was observed only in male subjects. Previous behavioral genetic studies have consistently suggested that alcohol and drug use in males was more determined by genetic factors, while that in female was more by environmental factors [27, 49]. In addition, male had markedly greater dopamine release than female in the ventral striatum in healthy volunteers, which could account for increased vulnerability of addictive disorders in male [50]. Likewise, gaming behaviors may be more influenced by genetic factors in males than in females due to the neurobiological differences between genders. Further studies are needed to determine different pathways to IGD between male and female.

We found that none of the polymorphisms were related with personality and temperament predispositions in IGD, except the association between the *del*+ genotype and high novelty seeking. Though age had significant interaction effect, this poses a possibility of the role of the − *141C* polymorphism on IGD. In vitro, the *del* allele-containing construct showed marked reduction in promoter activity, manifesting only 21–43% of the reporter gene expression level attributed to the *ins* allele-containing construct [16]. In addition, the *del*+ genotype showed differential neural response when performing the Go/No-go task in alcohol-abusing adults [51], suggesting the role of − *141C* polymorphism in important neurocognitive function. A recent study on the heritability of Internet addiction (IA) suggested that genetic factors accounted for 20–65% of a part of variance of specific IA factors such as personality factor self-directedness; they had negligible influence on generalized facets of IA [52]. Likewise, genetic factors may account for a certain part of the variance of temperament and personality traits observed in IGD. Though the *del*− genotype itself has been associated with alcohol dependence [20, 47, 53], the − *141C* polymorphism may have different roles in IGD possibly by mediating temperament traits.

Neither univariate and multivariate logistic regression analyses showed predictive value of polymorphisms of interest for the occurrence of IGD in adult population. Though we could not find the direct association of these polymorphisms with IGD, it is too hasty to draw final conclusion that these polymorphisms have no role in IGD. Given that genes interact with other genes as well as environmental factors and modify gene or protein expression levels even not altering DNA sequences, further researches using high-throughput technologies would give opportunities to explore these epistatic or epigenetic changes made in IGD.

This study had several limitations that should be noted. First, the sample size was relatively small given the possibility of false-positive findings in studies with small samples. However, this is a preliminary study to explore the role of the *DRD2* and *ANKK1* polymorphisms on IGD and would give an insight to the theme. Second, the IGD and control group had different mean age and gender distribution, which may confound the results. However, subgroup analyses in both genders and different age groups (age < 30 vs. age ≥ 30) showed comparable results with the original analyses. Third, psychiatric comorbidities such as depression or anxiety disorder and substance addictions were not screened. Nevertheless, since our sample was composed of non-clinical population and subjects with comparable intelligence levels, the proportion of psychiatric comorbidities is supposed to be small.

Despite the limitations, this study had some methodological strengths. First, our study focused on the adult population, which was a remarkable departure from previous studies that mainly focused on male adolescents. Since adolescents are susceptible to addictive disorder [46], the investigation of genetic predispositions in adult population would give more accurate perspectives by regressing out the contribution of characteristics of adolescents in the development of IGD. Second, we compared the IGD subjects with casual gamers. Considering that only a small portion of gamers become IGD out of casual gamers, this method had an advantage to investigate the neurobiological mechanisms that differentiate IGD from casual gamers. Third, the sample was homogeneous with respect to the ethnicity, education, and intellectual levels. By using interviews and objective measures, we excluded participants with neurological disorders and intellectual disabilities which may severely affect the personality and temperament traits.

## Conclusions

Our findings suggest that the genetic variations of the − *141C DRD2* may play a role on IGD via mediating specific symptoms or temperament traits in adult population, especially in male gender, though no direct association was observed. The neurobiological mechanisms of IGD may be different between adults and adolescents and between male and female. Further researches are necessary to investigate the interaction between genes and environmental factors and the differences across genders and age groups in neurobiological and psychological antecedents of IGD.

## Additional files

**Additional file 1.** Scatter plot with regression line of results of ANCOVA.

**Additional file 2.** Genotype and allele frequency for each criteria of DSM-5 IGD.

**Additional file 3.** Comparisons of personality and temperament between genotypes in Internet gaming disorder.
