# Supplementary material for: An association study of Taq1A ANKK1 and C957T and − 141C DRD2 polymorphisms in adults with internet gaming disorder: a pilot study
Source: Ann Gen Psychiatry. 2017 Dec 8;16:45. doi: 10.1186/s12991-017-0168-9 (PMC5721653; doi:10.1186/s12991-017-0168-9)

**Additional file 1. Scatter plot with regression line of results of ANCOVA**

Fig S1. Interaction effect of Age and NS in IGD and control groups (F=3.699, p=.030)


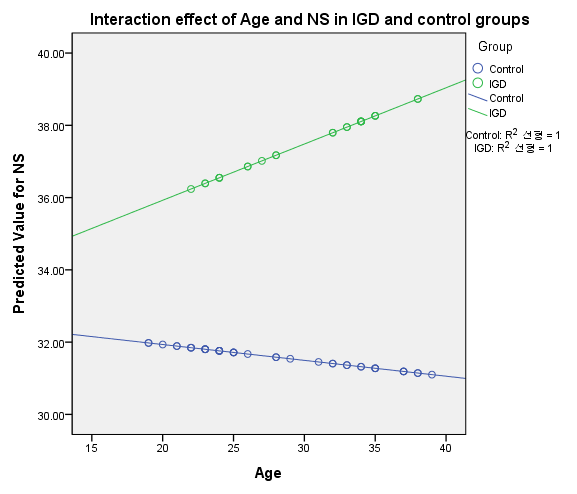


Fig S2. Interaction effect of Age and P in IGD and control groups (F=3.735, p=.029)


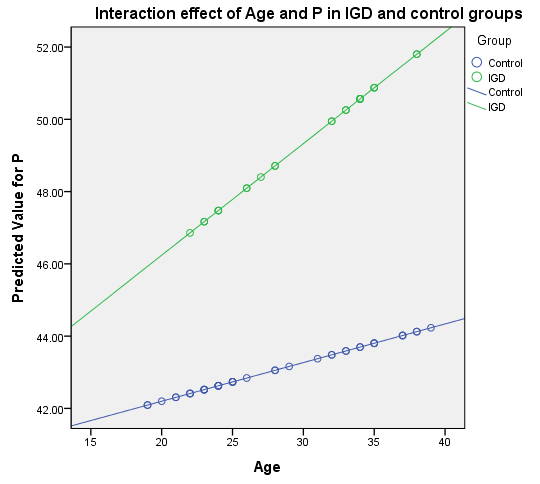


Fig S3. Interaction effect of Age and ST in IGD and control groups (F=5.285, p=.008)


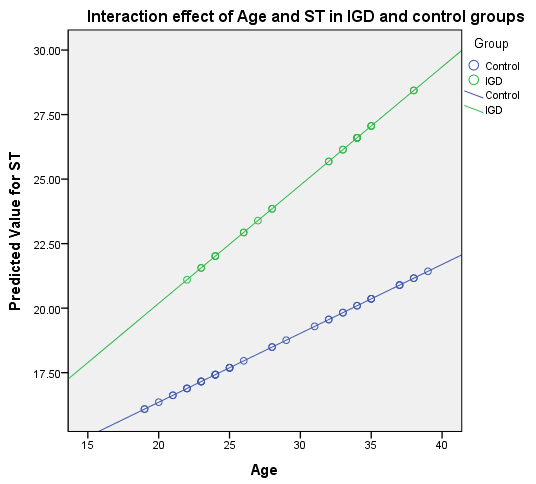


Fig S4. Interaction effect of Age and NS in del+ vs. del- (IGD subgroup (F=5.244, p=.013)


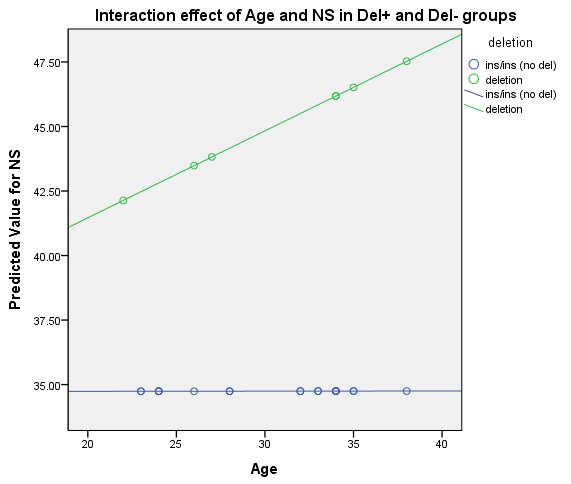

Supplement: Supplementary file 1 — Additional file 1. Scatter plot with regression line of results of ANCOVA. [file 12991_2017_168_MOESM1_ESM.docx]
