# Supplementary material for: An association study of Taq1A ANKK1 and C957T and − 141C DRD2 polymorphisms in adults with internet gaming disorder: a pilot study
Source: Ann Gen Psychiatry. 2017 Dec 8;16:45. doi: 10.1186/s12991-017-0168-9 (PMC5721653; doi:10.1186/s12991-017-0168-9)
Supplement: Supplementary file 2 — Additional file 2. Genotype and allele frequency for each criteria of DSM-5 IGD. [file 12991_2017_168_MOESM2_ESM.docx]

**Additional file 2. Genotype and allele frequency for each criteria of DSM-5 IGD**

| **S1. Nine criteria of DSM-5 IGD** | |
| --- | --- |
| G1 | Preoccupation with Internet games. |
| G2 | Withdrawal symptoms when Internet gaming is taken away. |
| G3 | Tolerance – the need to spend increasing amount of time engaged in Internet games. |
| G4 | Unsuccessful attempts to control the participation in Internet games. |
| G5 | Loss of interest in previous hobbies and entertainment |
| G6 | Continued excessive use of Internet games despite knowledge of psychosocial problems. |
| G7 | Has deceived family members, therapists, or others regarding the amount of Internet gaming. |
| G8 | Use of Internet games to escape or relieve a negative mood |
| G9 | Has jeopardized or lost a significant relationship, job, or educational or career opportunity because of participation in Internet games. |

| **S2. Allele and Genotype frequency of the Taq1A ANKK1 genotype in whole sample** | | | | | | |
| --- | --- | --- | --- | --- | --- | --- |
|  | A1A1 (n=27) | A1A2 (n=68) | | A2A2 (n=55) | χ^2^ | p |
| G1 | 6 (22.2%) | 22 (32.4%) | | 17 (30.9%) | .979 | .613 |
| G2 | 8 (29.6%) | 17 (25.0%) | | 19 (34.5%) | 1.338 | .512 |
| G3 | 10 (37.0%) | 24 (44.4%) | | 20 (36.4%) | .030 | .985 |
| G4 | 14 (51.9%) | 26 (40.6%) | | 24 (37.5%) | 1.498 | .473 |
| G5 | 12 (44.4%) | 23 (37.7%) | | 26 (42.6%) | 2.474 | .290 |
| G6 | 10 (37.0%) | 24 (35.3%) | | 20 (36.4%) | .030 | .985 |
| G7 | 13 (48.1%) | 28 (41.2%) | | 26 (47.3%) | .619 | .734 |
| G8 | 9 (33.3%) | 21 (30.9%) | | 23 (41.8%) | 1.649 | .438 |
| G9 | 2 (7.4%) | 16 (23.5%) | | 15 (27.3%) | 4.334 | .115 |
|  | A1+ (n=95) | | A1- (n=55) | | χ^2^ | p |
| G1 | 28 (29.5%) | | 17 (30.9%) | | .034 | .853 |
| G2 | 25 (26.3%) | | 19 (34.5%) | | 1.138 | .286 |
| G3 | 34 (35.8%) | | 20 (36.4%) | | .005 | .944 |
| G4 | 40 (42.1%) | | 24 (43.6%) | | .033 | .855 |
| G5 | 35 (36.8%) | | 26 (47.3%) | | 1.571 | .210 |
| G6 | 34 (35.8%) | | 20 (36.4%) | | .005 | .944 |
| G7 | 41 (43.2%) | | 26 (47.3%) | | .239 | .625 |
| G8 | 30 (31.6%) | | 23 (41.8%) | | 1.598 | .206 |
| G9 | 18 (18.9%) | | 15 (27.3%) | | 1.407 | .236 |

| **S3. Allele and Genotype frequency of the C957T DRD2 genotype in whole sample** | | | | | | |
| --- | --- | --- | --- | --- | --- | --- |
|  | TT (n=0) | TC (n=41) | | CC (n=129) | χ^2^ | p |
| G1 | 0 (0.0%) | 4 (19.0%) | | 41 (31.8%) | 1.395 | .238 |
| G2 | 0 (0.0%) | 5 (23.8%) | | 39 (30.2%) | .359 | .549 |
| G3 | 0 (0.0%) | 4 (19.0%) | | 50 (38.8%) | 3.046 | .081 |
| G4 | 0 (0.0%) | 9 (42.9%) | | 55 (42.6%) | .000 | .985 |
| G5 | 0 (0.0%) | 9 (42.9%) | | 52 (40.3%) | .049 | .826 |
| G6 | 0 (0.0%) | 7 (33.3%) | | 47 (36.4%) | .075 | .784 |
| G7 | 0 (0.0%) | 8 (38.1%) | | 59 (45.7%) | .427 | .514 |
| G8 | 0 (0.0%) | 6 (28.6%) | | 47 (36.4%) | .489 | .485 |
| G9 | 0 (0.0%) | 3 (14.3%) | | 30 (23.3%) | .847 | .357 |
|  | T+ (n=41) | | T- (n=129) | | χ^2^ | p |
| G1 | 4 (19.0%) | | 41 (31.8%) | | 1.395 | .238 |
| G2 | 5 (23.8%) | | 39 (30.2%) | | .359 | .549 |
| G3 | 4 (19.0%) | | 50 (38.8%) | | 3.046 | .081 |
| G4 | 9 (42.9%) | | 55 (42.6%) | | .000 | .985 |
| G5 | 9 (42.9%) | | 52 (40.3%) | | .049 | .826 |
| G6 | 7 (33.3%) | | 47 (36.4%) | | .075 | .784 |
| G7 | 8 (38.1%) | | 59 (45.7%) | | .427 | .514 |
| G8 | 6 (28.6%) | | 47 (36.4%) | | .489 | .485 |
| G9 | 3 (14.3%) | | 30 (23.3%) | | .847 | .357 |

| **S4. Allele and Genotype frequency of the -141C DRD2 genotype in whole sample** | | | | | | |
| --- | --- | --- | --- | --- | --- | --- |
|  | Del/Del (n=3) | Del/Ins (n=46) | | Ins/Ins (n=100) | χ^2^ | p |
| G1 | 1 (33.3%) | 13 (28.3%) | | 31 (31.0%) | .126 | .939 |
| G2 | 1 (33.3%) | 11 (23.9%) | | 32 (32.0%) | 1.011 | .603 |
| G3 | 1 (33.3%) | 15 (32.6%) | | 38 (38.0%) | .408 | .816 |
| G4 | 1 (33.3%) | 17 (37.0%) | | 46 (46.0%) | 1.167 | .558 |
| G5 | 2 (66.7%) | 16 (34.8%) | | 43 (43.0%) | 1.718 | .424 |
| G6 | 2 (66.7%) | 13 (28.3%) | | 39 (39.0%) | 2.799 | .247 |
| G7 | 1 (33.3%) | 20 (43.5%) | | 46 (46.0%) | .248 | .883 |
| G8 | 2 (66.7%) | 14 (30.4%) | | 37 (37.0%) | 1.884 | .390 |
| G9 | 2 (66.7%) | 7 (15.2%) | | 24 (24.0%) | 4.929 | .085 |
|  | Del+ (n=49) | | Del- (n=100) | | χ^2^ | p |
| G1 | 14 (28.6%) | | 31 (31.0%) | | .092 | .762 |
| G2 | 12 (24.5%) | | 32 (32.0%) | | .891 | .345 |
| G3 | 16 (32.7%) | | 38 (38.0%) | | .407 | .524 |
| G4 | 18 (36.7%) | | 46 (46.0%) | | 1.152 | .283 |
| G5 | 18 (36.7%) | | 43 (43.0%) | | .534 | .465 |
| G6 | 15 (30.6%) | | 39 (39.0%) | | 1.001 | .317 |
| G7 | 21 (42.9%) | | 46 (46.0%) | | .131 | .717 |
| G8 | 16 (32.7%) | | 37 (37.0%) | | .271 | .603 |
| G9 | 9 (18.4%) | | 24 (24.0%) | | .605 | .437 |
