# Supplementary material for: An association study of Taq1A ANKK1 and C957T and − 141C DRD2 polymorphisms in adults with internet gaming disorder: a pilot study
Source: Ann Gen Psychiatry. 2017 Dec 8;16:45. doi: 10.1186/s12991-017-0168-9 (PMC5721653; doi:10.1186/s12991-017-0168-9)
Supplement: Supplementary file 3 — Additional file 3. Comparisons of personality and temperament between genotypes in Internet gaming disorder. [file 12991_2017_168_MOESM3_ESM.docx]

**Additional file 3. Comparisons of personality and temperament between genotypes in Internet gaming disorder**

| **S5. Comparisons of personality and temperament between genotypes in Internet gaming disorder.** | | | | | | |
| --- | --- | --- | --- | --- | --- | --- |
|  | Taq1A genotype | | C957T genotype | | -141C genotype | |
|  | A1+ (n=38) | A1- (n=25) | T+ (n=6) | T- (n=57) | Del+ (n=19) | Del- (n=44) |
| Age (years old) | 31.87±6.593 | 32.28±6.554 | 33.67±6.593 | 31.86±6.556 | 32.05±5.671 | 32.02±6.926 |
|  | t=.243, p=.809 | | t=-.642 , p=.523 | | t=-.017 , p=.987 | |
| Weekday game hours | 2.88±1.56 | 2.00±.87 | 2.00±0.00 | 2.62±1.45 | 2.49±1.63 | 2.60±1.36 |
|  | t=-1.844 , p=.078 | | t=.593 , p=.559 | | t=.189 , p=.852 | |
| Weekend game hours | 4.21±1.99 | 3.22±1.64 | 3.50±.71 | 3.89±1.98 | 3.86±2.34 | 3.87±1.79 |
|  | t=-1.267 , p=.217 | | t=.277 , p=.784 | | t=.013 , p=.990 | |
| BSCS | 40.71±5.718 | 42.56±5.069 | 45.50±5.244 | 41.02±5.374 | 41.89±5.66 | 41.25±5.456 |
|  | t=1.318 , p=.193 | | t=-1.947 , p=.056 | | t=-.426 , p=.672 | |
| DII | 6.71±3.039 | 6.36±2.956 | 8.50±1.871 | 6.37±3.022 | 7.11±2.580 | 6.34±3.147 |
|  | t=-.453 , p=.652 | | t=-1.687 , p=.097 | | t=-.931 , p=.356 | |
| Novelty seeking | 36.652±2.140 | 39.212±2.950 | 32.129±6.180 | 37.989±1.782 | 44.71±9.050 | 34.86±6.863 |
|  | F=.489, p=.491 | | F=.830, p=.372 | | t=-2.973 , p=.007* | |
| Harm avoidance | 35.158±2.539 | 29.036±3.500 | 23.369±7.407 | 33.844±2.136 | 36.353±4.035 | 31.817±2.477 |
|  | F=1.987, p=.172 | | F=1.846, p=.187 | | F=.923, p=.347 | |
| Reward dependence | 43.162±2.435 | 48.027±3.357 | 48.940±7.237 | 44.505±2.087 | 46.042±3.886 | 44.406±2.357 |
|  | F=1.364, p=.255 | | F=.347, p=.562 | | F=.129, p=.722 | |
| Persistence | 47.641±2.068 | 52.900±2.850 | 54.773±6.191 | 49.019±1.785 | 49.003±3.364 | 49.630±2.040 |
|  | F=2.211, p=.151 | | F=.797, p=.381 | | F=.025, p=.875 | |
| Self-directedness | 50.212±2.311 | 50.600±3.186 | 55.373±6.637 | 49.927±1.914 | 48.354±3.562 | 51.080±2.160 |
|  | F=.010, p=.923 | | F=.621, p=.439 | | F=.428, p=.520 | |
| Cooperativeness | 53.278±1.781 | 59.141±2.455 | 53.934±5.575 | 55.422±1.608 | 52.309±2.893 | 56.412±1.755 |
|  | F=3.703, p=.067 | | F=.066, p=.800 | | F=1.469, p=.238 | |
| Self-transcendence | 25.489±2.533 | 23.854±3.492 | 23.424±7.386 | 25.048±2.130 | 27.494±3.902 | 23.976±2.366 |
|  | F=.142, p=.709 | | F=.045, p=.835 | | F=.594, p=.449 | |
| BSCS: Brief Self-Control Scale, DII: Dickman Dysfunctional Impulsivity scale.  *: p<.05. | | | | | | |
